# Supplementary material for: Sjögren syndrome/scleroderma autoantigen 1 is a direct Tankyrase binding partner in cancer cells
Source: Commun Biol. 2020 Mar 13;3:123. doi: 10.1038/s42003-020-0851-2 (PMC7070046; doi:10.1038/s42003-020-0851-2)
Supplement: Supplementary file 4 — Supplementary Information [file 42003_2020_851_MOESM4_ESM.pdf]

Sjögren syndrome/scleroderma autoantigen 1 is a direct Tankyrase binding partner in cancer cells. Harmonie Perdreau-Dahl *et al.*

SUPPLEMENTARY INFORMATION

SUPPLEMENTARY FIGURES

Supplementary Figure 1

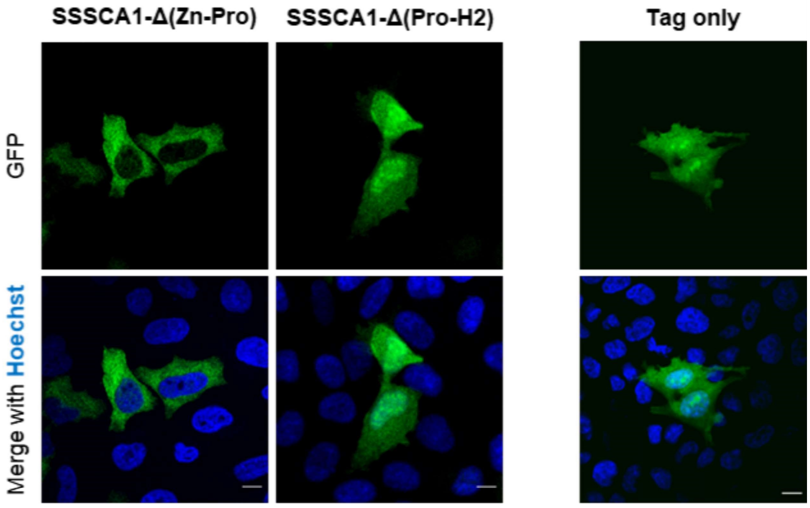

Supplementary Figure 1 SSSCA1 C-terminal domain behaves as a nuclear export signal. Additional confocal imaging of different GFP-tagged constructs of human SSSCA1 transiently expressed in HeLa cells and GFP-tag control. Scale bar: 10 µm.

Supplementary Figure 2

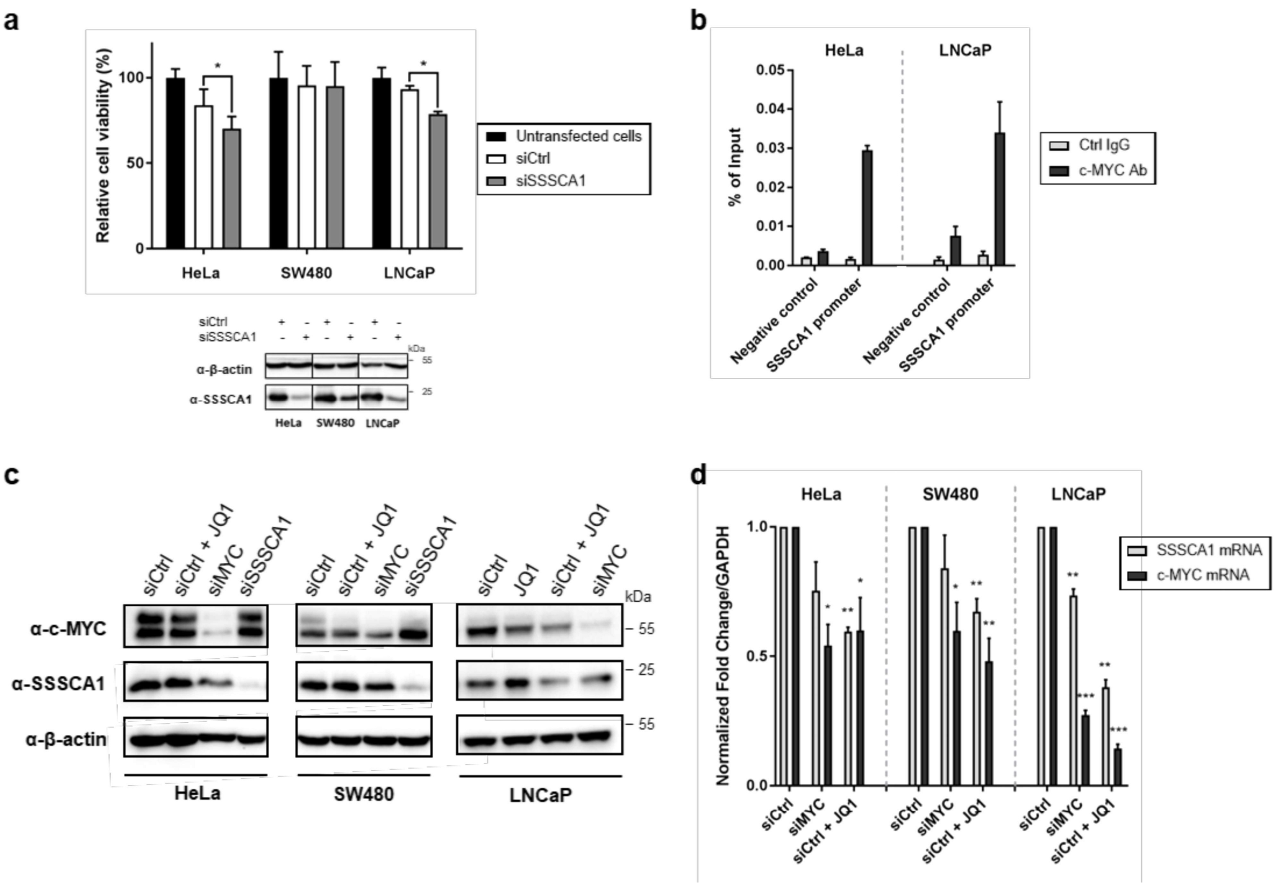

Supplementary Figure 2 SSSCA1 regulation in cancer cells. (a) Effects of SSSCA1 RNA silencing on cell viability. Cells were seeded in 96 wells plates and treated with siRNA for 72 hours. Cell viabilities were determined by MTT assay and plotted as a fraction relative to the non-transfected cells (black). *n* = 4; error bars, standard deviation. *Denotes statistically significant difference between control RNA silencing (siCtrl, white) and SSSCA1 RNA silencing (siSSSCA1, grey) with unpaired *t* test (*$p<0.05$). The efficiency of SSSCA1 silencing by siRNA at the protein level was controlled by immunoblotting against SSSCA1 (bottom panel). **(b)** c-MYC binds to the promoter of SSSCA1. Chromatin immunoprecipitation from HeLa and LNCaP cells with control (grey) or c-MYC (black) antibodies followed by qRT-PCR analysis of SSSCA1 promoter region. **(c)** c-MYC regulates SSSCA1 at the protein level. siRNA transfected cells were analyzed 72 hours post-transfection for protein levels by Western-Blotting. When indicated, 1 μM JQ1 drug was added to the cells during the last 24 hours. **(d)** c-MYC regulates SSSCA1 at the mRNA level. siRNA transfected cells were analyzed 72 hours post-transfection for mRNA levels (SSSCA1 in grey, c-MYC in black) by qRT-

PCR. When indicated, 1 µM JQ1 drug was added to the cells during the last 24 hours. $n = 3$; error bars, standard deviation. Significant statistical differences between siCtrl and (siMYC or siCtrl+JQ1) with unpaired *t* test are indicated (*p<0.05, **p<0.01, ***p<0.001).

Supplementary Figure 3

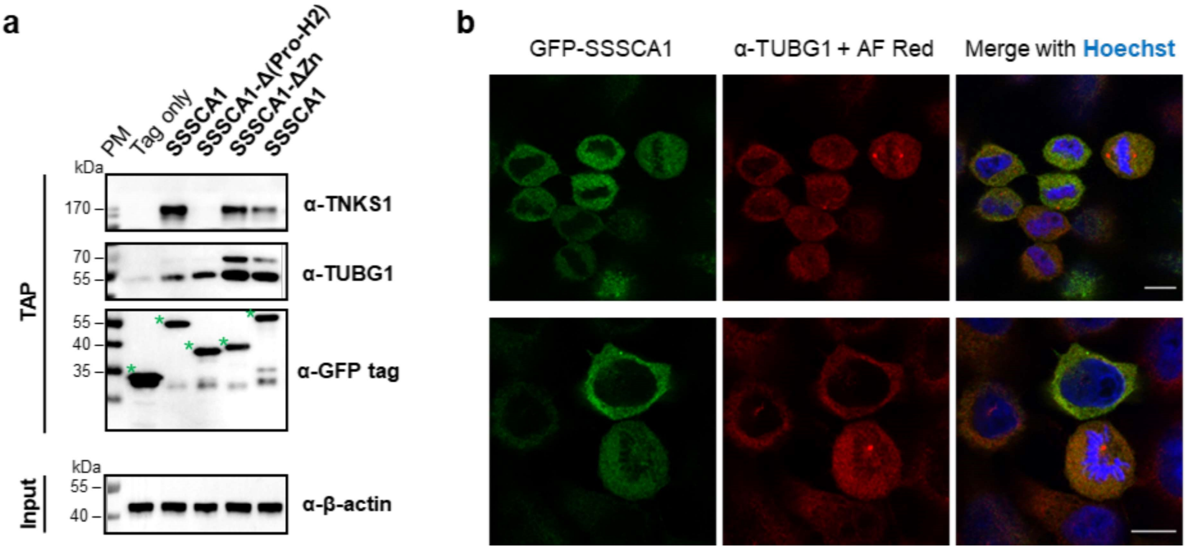

Supplementary Figure 3 TUBG1 is only a potential binding partner of SSSCA1. (a) Different constructs of human SSSCA1 were expressed in HeLa cells. After TAP, the binding of each construct to endogenous TNKS1 and TUBG1 was assessed by Western-blotting. Green stars indicate the constructs of interest. As compared to TNKS1, the pull down of TUBG1 does not show a clear preference to a specific domain in SSSCA1. **(b)** Confocal imaging of His-GFP-SSSCA1 and endogenous TUBG1 in HeLa cells. No clear co-localization between SSSCA1 and TUBG1 can be detected. Two representative examples are shown. AF, AlexaFluor. Scale bar: 10 µm.

Supplementary Figure 4

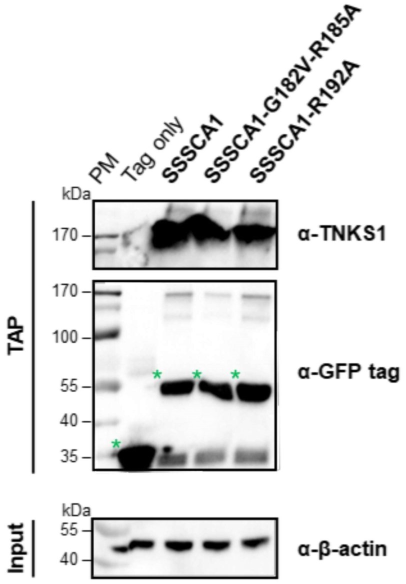

Supplementary Figure 4 Mutation of SSSCA1 arginine residues R185 and R192 do not alter the binding to Tankyrase 1. Different constructs of human SSSCA1 were expressed in HeLa cells. After TAP, the binding of each construct to endogenous TNKS1 was assessed by Western-blotting. Green stars indicate the constructs of interest. The mutation of arginine residues R185 and R192 into alanines do not alter the binding to TNKS1.

Supplementary Figure 5

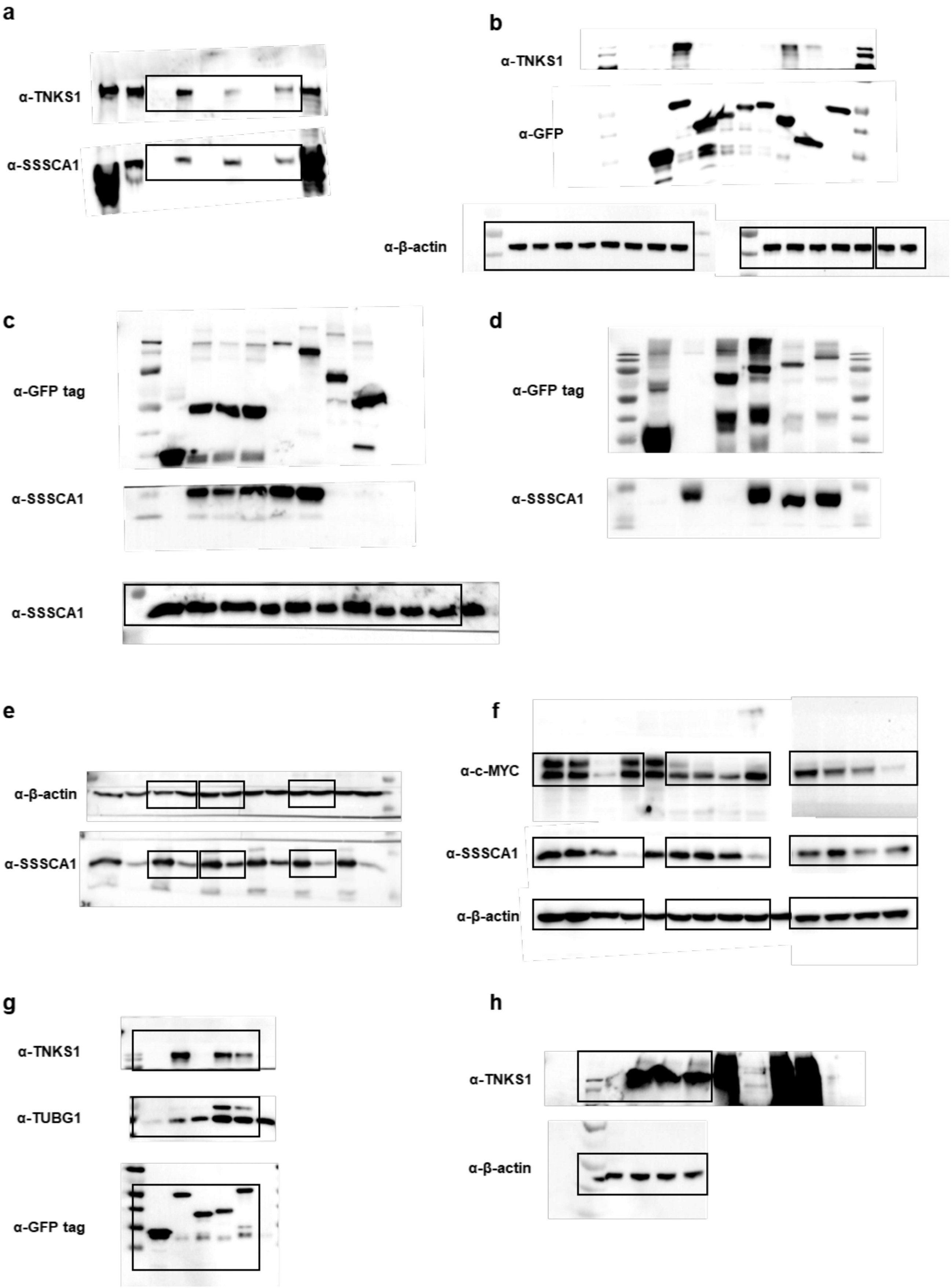

Supplementary Figure 5 Full blots.

SUPPLEMENTARY METHODS

siRNA transfection and Quantitative real-time PCR (qRT-PCR)

HeLa, LNCaP or SW480 cells were reverse transfected with siRNA and dispensed in 6-well (for Western Blotting analysis) or 24-well plates (for qRT-pCR analysis) for 72 hours. In some conditions, 1 µM BET bromodomain inhibitor JQ1, which has been shown to down regulate c-MYC expression, was added during the last 24 hours. Cells were then washed three times with PBS and cell pellets were frozen at -80°C.

Cell pellets from 6-well plates were lysed for 30 min, 4°C, in solubilization/lysis buffer (50 mM HEPES, pH 7.4, 150 mM NaCl, 5% glycerol, 0.5% NP40, 1 mM NaF, 1 mM Na_3VO_4, 0.5 mM TCEP, 1 µg/ml DNAse I, 1 mM PMSF and protease cocktail inhibitor (Roche)). To achieve a complete lysis, cells were sonicated for 4 cycles 30s ON/30 s OFF in a BioRuptor (Diagenode). Insoluble material was removed by centrifugation for 15 min at 15,000 x g, 4°C. The cleared lysates were normalized for protein concentration, reduced in 1x Laemmli buffer, boiled 3 min at 95°C and loaded on a 12% SDS-PAGE gel. Analysis by Western Blotting was afterwards done as described above.

Total RNA from 24-well plates' cell pellets was isolated using the RNeasy Mini Kit (Qiagen) and RNA were reverse transcribed with the SuperScript VILO cDNA synthesis kit (Invitrogen), following the manufacturer's instructions. Transcript levels were assessed using the 7900 HT Fast Real Time PCR System (AB). Real-time PCR was performed in 20 µl reactions consisting of 8 µl Primers Mix (1.25 µM for each primer), 10 µl 2x SYBR Green PCR Master Mix (Invitrogen), and 2 µl cDNA template. The thermocycling conditions used were 2 min at 50°C, 10 min at 95°C, followed by 40 cycles of 15 sec at 95°C and 1 min at 60°C. A dissociation stage was added at the end of the assay to check for contaminations and non-specific products amplification. All experiments were performed in triplicate. Gene expression analysis was performed using the Delta-Delta Ct approximation method with the housekeeping gene, GAPDH, for normalization. The following primer pairs were used (Sigma-Aldrich): SSSCA1 forward (CTCATGGGCGACTATCTGCT) and reverse (TTTGTCTTGGAGGAGGATCG), c-MYC forward (TACCCTCTCAACGACAGCAG) and

reverse (TCTTGACATTCTCCTCGGTG), TNKS1 forward (GGCAAACGTAAATGCAAAGG) and reverse (TGAGACCTCCATCATCACGA), GAPDH forward (TGAGGAGGGGAGATTCAGTG) and reverse (GTCAGTGGTGGACCTGACCT).

Chromatin immunoprecipitation (ChIP)

ChIP was performed using the Human c-MYC ExactaChIP Chromatin IP kit (R&D, ECP3696) with slight modifications to the manufacturer's protocol. Briefly, HeLa and LNCaP cells were crosslinked with 1% formaldehyde (Sigma, F8775). After scraping and cell lysis, chromatin was sheared to an average fragment size of 200–500 bp using a Bioruptor NextGen (Diagenode), cleared and diluted. Prior to overnight incubation with 5 µg anti-c-MYC/control IgG antibody, 1% of total chromatin was taken as input control. After harvesting the antibody-DNA complexes on a 4°C rotator for 1 h using 50 µl magnetic Streptavidin beads (R&D, MAG999), the DNA was eluted and reverse crosslinked for 16–20 h shaking at 65°C using 200 µl of a 1% SDS in $NaHCO_3$ solution. Subsequently, the DNA was purified using phenol-chloroform-isoamylalcohol extraction and reverse phase-lock tubes (5Prime, 2302830), precipitated with 100% EtOH, washed with 80% EtOH and finally resuspended in 60 µl Tris-HCl pH 8.0. ChIP qPCR was performed using the SYBR green master mix and same amplification conditions as mentioned above. Results are being displayed as "% of input" using the formula 2^(ct(Input)-ct(antibody)). The following primer pairs were used (Sigma-Aldrich): SSSCA1 forward (AAGCTTCTGTCCCCTGGATT) and reverse (CCTTAGCGTGGTCCTCAGAC), negative forward (AACTCCACATTTCCTAAGTGACC) and reverse (CCAACCCACACCAAGTACC).
