# Supplementary material for: Sjögren syndrome/scleroderma autoantigen 1 is a direct Tankyrase binding partner in cancer cells
Source: Commun Biol. 2020 Mar 13;3:123. doi: 10.1038/s42003-020-0851-2 (PMC7070046; doi:10.1038/s42003-020-0851-2)
Supplement: Supplementary file 5 — Description of Additional Supplementary Files [file 42003_2020_851_MOESM5_ESM.pdf]

Description of Additional Supplementary Files

File Name: Supplementary Data 1
Description: Mass Spectrometry data

File Name: Supplementary Data 2
Description: Fluorescence Polarization data
